# DOES STRESS MAKE YOU FEEL OLDER? A DAILY DIARY STUDY OF SUBJECTIVE AGING, STRESS, AND UPLIFTS IN TURKIYE

**DOI:** 10.1093/geroni/igad104.1535

**Published:** 2023-12-21

**Authors:** Reyyan Can, Shevaun Neupert

**Affiliations:** North Carolina State University, Raleıgh, North Carolina, United States; North Carolina State University, Raleigh, North Carolina, United States

## Abstract

Subjective aging, how one perceives their age, is associated with psychological, emotional, and social well-being. Adults’ subjective perceptions of their age are malleable and can fluctuate on a daily basis, influenced by various life events such as experiencing stress or uplifts. We conducted a 14-day daily diary study to assess how subjective age and ageist attitudes are predicted by daily stressful events and uplifts in Turkey/Turkiye. 68 Turkish participants aged 50 to 90 years (M= 57) responded to subjective age, ageist attitudes, stress, and uplifts questionnaires every day. Multilevel model analyses indicated that individuals perceived themselves as emotionally and physically older on days when they encountered more stressors and fewer uplifting events. Additionally, on those days, participants reported that they looked and behaved older than their chronological ages. Uplifts corresponded negatively to ageist attitudes; individuals who experienced more uplifting events during the day had fewer negative judgments of aging. Taking age as a subjective construct, these results suggest that having fewer stressors in daily life changes people’s feelings about their aging process. In addition, involvement in uplifts can buffer ageist attitudes on a daily basis.

